# Nutritional literacy in young and middle-aged patients with ischemic stroke: a mixed-methods study with IMB model-guided analysis

**DOI:** 10.3389/fnut.2026.1872887

**Published:** 2026-07-29

**Authors:** Zhenya Liu, Yanhong Chen, Cancan Chen, Weiwei Yang, Guifang Zhang

**Affiliations:** 1 Henan Provincial People's Hospital, Zhengzhou, China; 2 Peking Union Medical College Hospital, Beijing, China

**Keywords:** dietary adherence, IMB model, ischemic stroke, mixed-methods, nutritional literacy, young and middle-aged

## Abstract

**Background:**

Stroke incidence is rising among young and middle-aged adults, who bear major social and family responsibilities. Poor nutritional literacy is linked to unhealthy dietary behaviors and may increase stroke recurrence risk. However, evidence on nutritional literacy in this population remains limited, particularly under a validated theoretical framework.

**Aim:**

To evaluate nutritional literacy and explore its influencing factors among young and middle-aged patients with ischemic stroke using an explanatory mixed-methods design guided by the Information-Motivation-Behavioral Skills (IMB) model.

**Methods:**

A sequential explanatory mixed-methods study was conducted. Quantitative data were collected from 224 inpatients using the Chinese Nutrition Literacy Assessment Instrument (CHI-NLit). Univariate analysis was performed to identify associated factors. Qualitative data were obtained through semi-structured interviews with 16 purposively selected patients and analyzed using Colaizzi’s method under the IMB framework.

**Results:**

The mean nutritional literacy score was 18.24 ± 4.85 (scoring proportion 48%). 76.3% of patients scored below the adequacy threshold. Nutritional skill scores were significantly lower than cognitive scores; notably, 79.9% of patients scored zero in the food label calculation subscale. Univariate analysis showed that lower nutritional literacy was associated with middle age, low education, rural residence, low income, and manual occupation (all *p* < 0.05). Multivariable linear regression identified middle age (41–59 years, *β* = −0.239, *p* < 0.001) and high school education or above (vs. primary school or below, *β* = 0.351, *p* < 0.001) as independent determinants, whereas junior high school education was not significant (*p* = 0.067). Qualitative findings identified three core themes: fragmented information acquisition and poor knowledge-to-practice translation, complex motivation involving family responsibility and negative emotions, and multiple behavioral skill deficits including food label interpretation, workplace social dilemmas, and dynamic self-efficacy.

**Conclusion:**

This mixed-methods study reveals that nutritional literacy is insufficient among young and middle-aged patients with ischemic stroke, with particularly severe deficits in practical skills. The IMB model effectively identifies barriers across information, motivation, and behavioral skills domains. The integration of quantitative and qualitative findings provides a comprehensive evidence base for designing targeted interventions. However, longitudinal and interventional studies are needed to determine whether improving nutritional literacy reduces stroke recurrence or improves clinical outcomes.

## 1 Introduction

Stroke is a leading chronic non-communicable disease that severely threatens public health in China. According to the American Heart Association, the number of stroke patients worldwide in 2019 was 101.5 million, with approximately 6.6 million deaths attributed to stroke. The combined direct and indirect economic burden of stroke was approximately $49.8 billion, causing a serious burden on the whole society and family (1). Ischemic stroke accounts for about 87% of global epidemiology of stroke (2). China is located in the world’s worst stroke incidence area, the number of stroke patients ranks first in the world, and the incidence and prevalence of ischemic stroke account for a large proportion and the burden is heavy (3, 4). The vigorous promotion of stroke identification education and emergency rescue systems, together with the optimization of stroke green channel services, enables ischemic stroke patients to receive timely treatment in the acute phase (3). Moreover, continuous advances in vascular recanalization technology and the development of surgical methods further help prevent stroke-related disability and functional impairment (3).

However, it is worth noting that stroke has a high recurrence rate. The factors influencing stroke recurrence encompass age, hypertension, diabetes mellitus, dyslipidemia, and cardiovascular disease (5, 6). Poor adherence to secondary prevention medications is significantly associated with increased risk of stroke recurrence, highlighting the importance of risk factor control (7).

With the development of economy and society, while material living standards have improved, unhealthy lifestyles and dietary habits have led to unreasonable dietary structures, and the risk factors for stroke have increased significantly. Consequently, this leads to a rising incidence of stroke and a clear trend toward younger age of onset (8). According to the 2021 survey, patients aged 18–50 years account for 10% of all stroke patients globally (1). According to the survey data of “China Stroke Prevention and Treatment Report” (3), among the newly identified high-risk groups in 2020, the standardized percentage of individuals aged 40 to 64 years is 71.80%. As a strong psychological stressor, a stroke seriously hinders young and middle-aged people from undertaking work and participating in various social activities (9). The middle-aged and young people are regarded as the labor force, they are the main labor force of society, the pillars of families, the backbone of the society and the country. Once a stroke occurs, they are no longer able to assume their social roles and family function, affecting not only their work and quality of life but also placing a heavy burden to the whole family and society. Young and middle-aged individuals often neglect their own health due to high work pressure and fast-paced lifestyles, making them more susceptible to unhealthy behaviors, including poor dietary patterns, overweight and obesity, smoking, alcohol consumption, and physical inactivity. These factors not only contribute to the onset of ischemic stroke but may also increase the risk of disease recurrence (10).

The factors that form unhealthy lifestyles are complex. They are not only affected by social economy and material environment, but also vary according to individual abilities and skills. If the individual’s health knowledge and other information support is insufficient, the lack of skills to deal with health problems will affect their health behavior (11). Nutritional literacy refers to the ability of people to obtain, understand, process basic nutritional information and services and make correct nutritional decisions to maintain and promote their own health (12). Nutritional literacy is a significant predictor of anthropometric measurements, healthy lifestyle behaviors, such as dietary habits, as well as quality of life (1). A higher level of nutritional literacy can help individuals obtain correct nutritional information to improve dietary quality (13), optimize dietary structure, and effectively prevent and manage chronic diseases (1). Furthermore, enhanced nutritional literacy empowers individuals to make informed choices, improve their adherence to dietary recommendations, enhance self-efficacy, and consequently reduce the incidence of complications while lowering healthcare expenditures (14).

However, research on nutritional literacy in stroke patients remains limited, particularly for young and middle-aged populations—a group facing unique challenges due to their societal roles and lifestyle patterns. Existing studies have primarily employed quantitative designs to describe nutritional literacy levels, leaving the underlying mechanisms and contextual factors that shape patients’ nutritional literacy largely underexplored. Moreover, the absence of a theoretical framework to systematically analyze these influencing factors hinders the development of targeted interventions to improve nutritional literacy and reduce stroke recurrence risk in this population.

To address the gaps in theoretical framework construction and qualitative evidence accumulation in existing research, this study introduces the Information–Motivation–Behavioral Skills (IMB) model as its theoretical framework. The IMB model posits that the formation and maintenance of health behaviors rely on the interplay of three core components: information (knowledge related to health behaviors), motivation (individual attitudes, subjective norms, and intention to change), and behavioral skills (practical competencies and self-efficacy required to perform health behaviors).

In the present study, these three dimensions correspond clearly to nutritional literacy and are operationalized using a mixed methods approach. The information dimension (post-stroke nutritional knowledge, dietary principles, and awareness of nutritional risks) is assessed via the quantitative survey, while qualitative interviews explore the reliability and completeness of patients’ information sources. The motivation dimension (adherence motivation, health beliefs, family support, and emotional influences) is examined through statistical analyses of its associations with nutritional literacy scores, complemented by in-depth interviews that contextualize the emergence and fluctuation of motivation over time. The behavioral skills dimension (food label comprehension, portion estimation, healthy cooking, and coping strategies for social situations) is quantified using scale-based assessments, while interview data elucidate how skill limitations impede the translation of nutritional knowledge into actual dietary behavior. Together, the IMB model fully covers the continuum of nutritional literacy from cognition and attitudes to practice among young and middle-aged stroke survivors, rendering it an appropriate and operationalizable theoretical framework for this study.

Previous research supports the application of the IMB model. For instance, Wang et al. (15) used the IMB model to explore factors influencing exercise rehabilitation behavior in hypertensive patients, identifying three themes (Information, Motivation, Behavioral Skills) and six sub-themes. Che et al. (16) used the model’s core elements as independent variables to study treatment adherence in hemodialysis patients, finding that self-efficacy, social support, and self-management positively influenced adherence. Similarly, Kim et al. (17) investigated factors influencing self-management in stroke patients based on the IMB model.

Therefore, evaluating the nutritional literacy status of young and middle-aged ischemic stroke patients constitutes a crucial initial step in intervention studies and represents a significant advancement in preventing stroke recurrence within this patient population. Additionally, the Outline of the “Healthy China 2030” Plan and the National Nutrition Plan (2017-2030) list the improvement of the nutritional literacy of the whole people as an important health development goal. To accurately assess nutritional literacy, a valid and reliable measurement tool is essential. The Nutrition Literacy Assessment Instrument (NLit) has been widely validated across diverse populations and is notable for its comprehensive evaluation of both cognitive and practical skills (18). The Chinese version (CHI-NLit), adapted for patients with chronic conditions (19), was therefore employed in this study.

Therefore, this study aims to examine the current status of nutritional literacy among young and middle-aged patients with ischemic stroke, thereby providing a foundation for subsequent intervention research. There is no unified definition of nutritional literacy, and different scholars have provided varying definitions (20). In summary, the concept originated from the broader framework of “Health Literacy” with a specific emphasis on the domain of diet and nutrition. Nutritional literacy encompasses two primary dimensions: cognitive understanding and practical skills. Cognitive understanding involves knowledge and concepts pertaining to food nutrition, while practical skills encompass the abilities required for acquiring and planning food, selecting food, preparing meals, and consuming food (21).

To fill this research gap, this study adopted an explanatory sequential mixed methods design. Its advantages are threefold. First, the quantitative phase (questionnaire survey) identified the overall status and key influencing factors of nutritional literacy deficiencies, while the qualitative phase (in-depth interviews) elucidated the underlying mechanisms behind the quantitative findings, thereby achieving both description (“what”) and explanation (“why”). Second, quantitative data captured correlations among variables, whereas qualitative data revealed patients’ authentic experiences and behavioral logic; the triangulation of these two approaches enhanced the robustness and clinical credibility of the study’s conclusions. Third, the mixed methods design enabled the incorporation of patients’ subjective perspectives into intervention development, thereby improving the specificity and acceptability of future intervention strategies.

A cross-sectional survey using the CHI-NLit scale was first conducted to assess the nutritional literacy status of 224 young and middle-aged ischemic stroke patients. Subsequently, semi-structured interviews guided by the IMB model were conducted with 16 purposively selected patients to explore the influencing factors of their nutritional literacy in depth. The integrated findings were synthesized into core themes, elucidating the dynamic interplay of information, motivation, and behavioral skills in the development of patients’ nutritional literacy. This study provides both a novel theoretical perspective and empirical evidence to inform targeted interventions aimed at improving nutritional literacy in this population. Importantly, the IMB model was applied in this study to explain the mechanisms linking nutritional literacy to dietary adherence in young and middle-aged ischemic stroke patients, moving beyond simple correlation analysis.

## 2 Materials and methods

### 2.1 Study design and population

**Figure 1:**
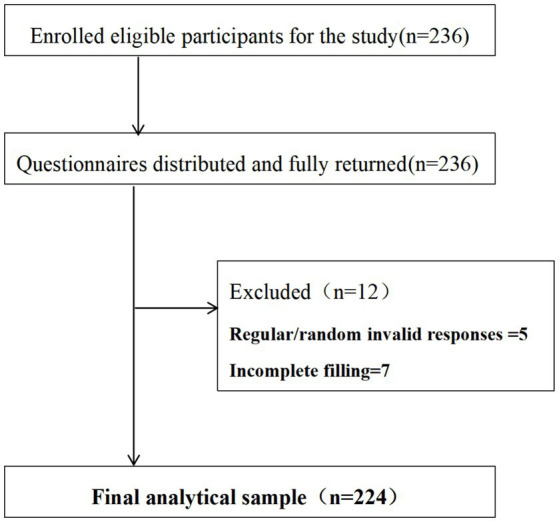
Participant screening flowchart for the quantitative study.

This study uses a sequential mixed research design. The investigation on the nutritional literacy status of middle-aged and young patients with ischemic stroke is a single-center, cross-sectional survey. A convenience sampling method was employed to recruit 236 inpatients from the stroke nursing unit of a tertiary teaching hospital in Henan Province, between March 2025 and July 2025. The questionnaire was mainly distributed by 2 nurses with master’s degree. Both nurses specialized in stroke care, and they all underwent unified training prior to the investigation. First, with the approval of the nursing administrator, 2 nurses went to the ward to recruit participants one-on-one. They explained to the participants that the objectives of the study, obtained consent, and detailed the procedures involved. Following the acquisition of signed consent forms from the participants, the questionnaires were officially distributed. Participants could complete the questionnaire in two ways: either on-site or by returning it within 2 days. If unable to complete it independently due to low vision or literacy, researchers read the questions and options using standardized verbal instructions, recorded the participant’s answers, and selected the responses. Inclusion criteria: (1) Patients met the diagnostic criteria for ischemic stroke according to the Chinese Classification of Cerebrovascular Diseases (2015) (22) and were confirmed as ischemic stroke through brain CT or MRI examination; (2) Age between 18 and 59 years, classified as young and middle-aged adults according to previous research (9); (3) Participants were clinically stable and able to communicate without barriers; (4) Participants had informed consent and were willing to participate in this study. Exclusion criteria: (1) Participants had cognitive impairment or mental illness; (2) Participants had serious complications or other serious medical conditions that prevented them from cooperating with the questionnaire. The sample size was determined by the current situation study sample size calculation formula *N* = (*Z*^2^_1-*α*/2_pq)/*d*^2^ (23), *p* represents nutritional literacy, and it is found in literature that *p* = 0.653, *q* = 1-*p*, when *d* = 0.1 × *p*, *a* = 0.05, Z1-*α*/2 = 1.96. Based on the above information, a minimum sample size of 205 was required. Considering the 15% invalid questionnaire, the final sample size was 236. All 236 distributed questionnaires were completely returned, corresponding to a questionnaire response rate of 100.0%. After exclusion of 12 unqualified questionnaires, 224 valid samples were included for subsequent statistical analysis. Details are presented in Figure 1.

For the qualitative component of this study, which aimed to explain the quantitative findings and to elucidate the underlying mechanisms and lived experiences contributing to inadequate nutritional literacy among patients, we adopted a descriptive phenomenological approach rooted in Husserl’s philosophy. We employed purposive sampling for participant recruitment. From March to July 2025, young and middle-aged patients with ischemic stroke who participated in the investigation earlier were selected as participants for semi-structured interviews.

Inclusion Criteria:

(1) Confirmed diagnosis of ischemic stroke, meeting the diagnostic and age criteria (18 ~ 59 years) from the prior questionnaire survey.
(2) Completion of the previous nutritional literacy status questionnaire.
(3) Possession of basic expressive ability to clearly and coherently describe personal experiences and perceptions.
(4) Clear consciousness, unimpaired communication, and voluntary participation with signed informed consent for the interview.

Exclusion Criteria:

(1) Presence of severe cognitive impairment, psychiatric disorders, or neurological sequelae (e.g., severe aphasia, significant hearing impairment) that hinder normal communication.
(2) Unstable medical condition or acute phase of illness, making it difficult to complete an in-depth interview lasting approximately 30–60 min.
(3) Concurrent participation in other interventional studies that may influence the outcomes of this research.

To operationalize these exclusion criteria, cognitive impairment was screened using the Mini-Mental State Examination (MMSE), with a cutoff of <24 indicating possible impairment, and such patients were excluded (24). Communication disorders, including aphasia, were assessed using the Chinese version of the Western Aphasia Battery (WAB) or by clinical judgment of a speech-language therapist (25). Patients with severe aphasia who were unable to understand simple questions or express basic needs were excluded.

The sample size is determined based on the saturation of coding or themes when conducting the data analysis (26). Data saturation was deemed reached after the 16th interview, as no new codes were identified. This was confirmed by interviewing two additional eligible patients (interviews 17 and 18), during which no new themes emerged. Data from these two confirmatory interviews were not included in the final analysis, as they served only to verify saturation. Thus, 16 participants constituted the final analytic sample.

#### 2.1.1 Integration of the mixed methods design

This study adopted an explanatory sequential mixed methods design, which consisted of two consecutive phases.

Phase 1 (Quantitative-led): A cross-sectional questionnaire survey was conducted (*N* = 224) with the following objectives: (1) to describe the overall level and achievement rate of nutritional literacy; (2) to examine differences across sociodemographic subgroups; and (3) to preliminarily identify weaknesses in the information and behavioral skills dimensions of the IMB model. The results of the quantitative phase guided participant selection for the subsequent qualitative phase.

Phase 2 (Qualitative explanatory): Based on the findings from Phase 1, purposive maximum variation sampling was used to select 16 patients for semi-structured interviews, covering a range of ages, educational levels, income levels, and nutritional literacy scores. The interview objectives were: (1) to explain why nutritional skills scores were significantly lower than knowledge scores; (2) to reveal the psychological mechanisms underlying quantitative results; and (3) to explore behavioral barriers not measurable by scales.

Data Integration Strategy: Data integration was guided by the IMB model. In the Results section, quantitative data (Tables 1–3) and qualitative themes (Section 3.2) are presented separately. In the Discussion section, a joint display approach was employed to juxtapose quantitative findings and qualitative themes for triangulation, systematically examining their consistency and complementarity.

**Table 1:** General characteristics of study participants and univariate analyses of total nutrition literacy score (*N* = 224).

Variable/Group		*N* (%)	Nutrition literacy			
			*M* ± SD	*t*/*F*	*P*	*Post hoc*
Age (years)	Young (18 ~ 40)	39(17.41)	21.82 ± 4.93	5.389	0.001	–
	Middle-aged (41 ~ 59)	185(82.59)	17.48 ± 4.49			
Gender	Male	142(63.4)	18.58 ± 4.88	1.38	0.167	–
	Female	82(36.6)	17.65 ± 4.77			
Education Level	Primary school or below^1^	50(22.3)	15.10 ± 3.72	31.225^##^	0.001	1<2<3
	Junior high school^2^	90(40.2)	17.34 ± 3.72			
	High school or above^3^	84(37.5)	21.06 ± 5.03			
Occupational Status	Employed^1^	86(38.4)	20.45 ± 4.91	19.249^#^	0.001	1>2 1>3
	Farming/Laborer^2^	99(44.2)	16.34 ± 4.14			
	Retired^3^	39(17.4)	18.15 ± 4.39			
Medical expense payment mode	New Rural Cooperative Medical Scheme ^1^	136(60.7)	16.79 ± 4.44	17.652^#^	0.001	1<2 1<3
	Urban Medical Insurance^2^	11(4.9)	20.09 ± 3.75			
	Employee Medical Insurance^3^	77(34.4)	20.52 ± 4.75			
Living Arrangement	Living alone	11(4.9)	18.91 ± 4.85	0.471	0.638	–
	With spouse, children, or parents	213(95.1)	18.20 ± 4.86			
Smoking Status	No	111(49.6)	18.15 ± 5.29	−0.255	0.779	–
	Yes	113(50.4)	18.32 ± 4.40			
Alcohol Consumption History	No	99(44.2)	18.13 ± 5.09	−0.289	0.773	–
	Yes	125(55.8)	18.32 ± 4.67			
Current Residence	Rural	101(45.1)	16.88 ± 4.52	3.911	0.001	–
	Urban	123(54.9)	19.35 ± 4.83			
Disease Duration (years)	<1	163(72.8)	18.23 ± 5.02	0.038	0.963	–
	1–3	41(18.3)	18.37 ± 4.64			
	>3	20(9.0)	18.00 ± 3.99			
Monthly *Per Capita* Household Income (RMB)	≤2000^1^	96(42.9)	16.25 ± 4.21	17.250^#^	0.001	1<2 1<3
	2001 ~ 4000^2^	76(33.9)	19.24 ± 5.00			
	≥4001^3^	52(23.2)	20.44 ± 4.39			
Frequency of Stroke Hospitalization	1	126(56.3)	18.44 ± 5.17	0.253	0.777	–
	2	62(27.7)	18.03 ± 4.52			
	≥3	36(16.1)	17.89 ± 4.26			
Hypertension	No	116(51.8)	18.60 ± 5.12	1.180	0.239	–
	Yes	108(48.2)	17.84 ± 4.53			
Coronary heart disease	No	206(92.0)	18.15 ± 4.81	−0.899	0.370	–
	Yes	18(8.0)	19.22 ± 5.34			
Diabetes	No	167(74.6)	18.25 ± 4.89	0.047	0.936	–
	Yes	57(25.4)	18.21 ± 4.76			

The superscript letters 1–3 are designated to compare each sub-variable when conducting the post hoc test. ## indicates homogeneity of variance verified by Levene’s test (*P* > 0.05), with Bonferroni test adopted for post-hoc pairwise comparisons; # indicates heterogeneous variance (*P* < 0.05), with Tamhane’s T2 test used for post-hoc multiple comparisons. Two-group comparisons were performed using independent-samples *t*-test or Welch’s *t*-test without *post hoc* analysis.

**Table 2:** Scores of the CHI-NLit dimensions.

Dimension	Item Count	Score	Proportion of scores
Total CHI-NLit	38	18.24 ± 4.85	48.00%
Knowledge dimensions			
Nutrition and Health	7	3.49 ± 1.58	49.86%
Energy Sources in Food	6	2.56 ± 1.66	42.67%
Food Groups	4	2.22 ± 0.80	55.50%
Skills dimensions			
Household Food Measurement	6	3.03 ± 1.67	50.50%
Consumer Skills	9	6.14 ± 1.57	68.22%
Food Label and Numeracy	6	0 (0, 0)	13.17%

Proportion of scores = actual average score/full score × 100%. The Food Label and Numeracy dimension showed non-normal distribution, described as median (P25, P75); 79.9% of participants obtained zero score in this subscale.

**Table 3:** Assignment method of independent variables.

Independent variables	Assignment method
Education level	Primary school and below = (0, 0, 0); Junior high school = (0, 1, 0); Senior high school and above = (0, 0, 1)
Occupational Status	Farming = (0, 0, 0); Retired = (0, 1, 0); Employed = (0, 0, 1)
Medical expense payment mode	New Rural Cooperative Medical Scheme = (0, 0, 0); Urban medical insurance = (0, 1, 0); Employee medical insurance = (0, 0, 1)
Age (years)	Younger age group (18 ~ 40) = 0; Older age group (41 ~ 59) = 1
Current Residence	Rural = 0; Urban = 1
Monthly per capita household income	≤2000 = 1; 2001 ~ 4,000 = 2; ≥4,001 = 3 Original value input (continuous variable)

### 2.2 Measurements

#### 2.2.1 General information questionnaire

The general information questionnaire was designed based on the research objectives and content, and comprised 12 items. It included gender, age, ethnicity, education level, monthly family income, mode of medical payment, current residence, BMI, smoking history, alcohol consumption history, disease duration, and presence of other underlying medical conditions.

#### 2.2.2 CHI-NLit

The Chinese version of the Nutrition Literacy Assessment Instrument (CHI-NLit): The original scale was developed by Gibbs et al. (18) and further revised in 2018. The CHI-NLit was translated and adapted by Chen et al. (19), comprising six dimensions (Nutrition and Health, Energy Sources in Food, Household Food Measurement, Food Label and Numeracy, Food Groups, and Consumer Skills) with a total of 38 items. Each correct response is assigned 1 point, while incorrect responses are assigned 0 points. The total score ranges from 0 to 38, with a cutoff value of 21.5 used to distinguish between low and adequate levels of nutritional literacy. The scale has demonstrated good reliability and validity in previous studies (27). In the present study, the Chinese version was employed to assess patients’ nutritional literacy. Based on conceptual analysis, the dimensions “Nutrition and Health,” “Energy Sources in Food,” and “Food Groups” pertain to nutritional knowledge, while the remaining three dimensions reflect nutritional skills. In this study, the overall Cronbach’s *α* coefficient for nutritional literacy was 0.803, while the coefficients for individual dimensions ranged from 0.821 to 0.960. Of note, the recommended cutoff value of 21.5 points for the CHI-NLit was originally established and validated in general chronic disease populations but has not yet been specifically validated in patients with ischemic stroke. Therefore, this cutoff was used exploratorily in the present study to preliminarily classify nutritional literacy levels, and findings based on this threshold should be interpreted with caution pending further validation in stroke-specific populations.

#### 2.2.3 The interview guide

We collected data through semi-structured interviews (SSI). The interview guide was developed by the researchers. Based on the research objectives and in combination with the actual clinical situation, the researchers conducted a literature review and, based on the framework of Information-Motivation-Behavioral Skills Model, formulated the interview outline from three aspects: information, motivation and behavioral skills. To ensure the effectiveness of the interview guide, the nursing managers from the stroke department and other research team members reviewed it and unanimously agreed with its content. We conducted two pre-interviews of the interview guide to verify the clarity of the interview questions and made necessary modifications, and ensured that the researchers were familiar with the interview techniques before the formal data collection stage. After this verification process, data collection began. The interview guide was as follows: ① What are the primary sources through which you obtain dietary guidance following a stroke, and to what extent do you find such information practicable in daily living? ② In implementing these dietary recommendations, what constitute the most significant uncertainties or challenges you encounter, and how do you typically address them? ③ Could you please share what exactly was the driving force that enabled you to persist on the path of maintaining a healthy diet after suffering a stroke? Also, what were the situations or thoughts that might have hindered your progress? And in this process, what roles did your family or friends play? ④ In daily practice, what is the greatest challenge to dietary adherence? When facing challenges such as social situations, emotions, or time constraints, how do you usually handle them? ⑤ Could you share a particularly rewarding or frustrating experience in the field of diet management? After that experience, did your perception of your ability to stick to a healthy diet change?

Two researchers who received formal training in qualitative methodology conducted all individual, face-to-face, semi-structured interviews. Before the interview, the researcher scheduled a time and quiet, comfortable place to avoid interruptions. The interviewer explained the study’s goals, secured informed consent, and began audio recording. Throughout the process, the interviewer used techniques like probing and reflecting to help participants elaborate on their views without being led, and also noted non-verbal information such as expressions, gestures, and emotional reactions. The interviews were dynamic; we were able to use our creativity based on the different participants, and they provided flexibility to adjust the questions to said participants. Each participant was assigned a unique identifier (P1 to P16) based on the order of interview completion. These identifiers are used throughout the results section to ensure traceability and protect anonymity.

#### 2.2.4 IMB model dimension-specific measurements and data integration strategy

To systematically operationalize the three core dimensions of the IMB model, this study adopted a combined measurement strategy. Information dimension: This dimension was assessed using three subscales of the CHI-NLit: “Nutrition and Health,” “Energy Sources in Food,” and “Food Groups,” which evaluate patients’ post-stroke dietary knowledge. In data analysis, scores from this dimension were used to describe the overall status of patients’ knowledge deficits and to identify sociodemographic characteristics associated with knowledge levels through univariate analysis. Motivation dimension: Motivation data were obtained exclusively through the motivation module of the semi-structured interview guide (Questions ③, ④, and ⑤), which explored patients’ adherence motivation, health beliefs, family support, and emotional influences. Qualitative analyses revealed the specific contexts and psychological processes underlying the emergence, maintenance, and dissipation of dietary adherence motivation. Behavioral skills dimension: Skill levels were assessed using three subscales of the CHI-NLit: “Household Food Measurement,” “Food Label and Numeracy,” and “Consumer Skills.” Concurrently, Questions ①, ②, and ④ of the interview guide specifically explored patients’ practical abilities in cooking, food label use, and coping with workplace social situations. Quantitative data were used to identify the prevalence and severity of skill deficits, while qualitative data elucidated the specific manifestations and functional consequences of these skill limitations in daily life.

### 2.3 Data analyses

#### 2.3.1 Quantitative data analysis

All statistical analyses were conducted with IBM SPSS Statistics 26.0 (IBM Corp., Armonk, NY, United States). The total nutritional literacy score was treated as a continuous dependent variable. Normality of score distribution was verified via the Shapiro–Wilk test separately for each subgroup stratified by all categorical covariates (age, gender, education level, occupational status, medical expense payment mode, living arrangement, smoking history, alcohol consumption history, residential location, disease duration, monthly per capita household income, frequency of stroke hospitalization, hypertension, coronary heart disease, and diabetes), rather than solely for the full pooled sample. All subgroup datasets satisfied the normality assumption (*p* > 0.05). Prior to all parametric statistical tests, Levene’s test was implemented to check homogeneity of variance across comparison sets.

##### 2.3.1.1 Univariate analyses

Two-group comparisons were conducted using the independent-samples *t*-test under homogeneous variance (Levene’s *p* > 0.05), whereas Welch’s *t*-test was adopted when the variance assumption was violated (Levene’s *P* < 0.05); such two-group covariates included gender, residential location, living arrangement, smoking status, alcohol consumption history, hypertension, coronary heart disease, and diabetes. For variables consisting of three or more subgroups (education level, occupational status, medical expense payment mode, disease duration, monthly per capita household income, and stroke hospitalization times), one-way ANOVA was carried out. Where ANOVA yielded significant between-group differences, post-hoc pairwise comparisons were implemented accordingly: Bonferroni correction was employed for groups with equal variances (Levene’s *p* > 0.05), and Tamhane’s T2 test was selected for datasets with heterogeneous variances (Levene’s *p* < 0.05).

##### 2.3.1.2 Multivariable analyses

All covariates reaching statistical significance at (*p* < 0.05) in univariate analyses were incorporated into a multiple linear regression model via the enter selection method, where total nutrition literacy score served as the continuous dependent outcome. The independent predictors comprised age, education level, occupational status, medical expense payment mode, residential location, and monthly per capita household income. Variance inflation factor (VIF) was calculated to detect multicollinearity; VIF > 5 was defined as evidence of severe multicollinearity. The Durbin–Watson statistic was applied to examine residual autocorrelation, while adjusted *R*^2^and the global *F*-test were used to quantify overall model goodness-of-fit. For all statistical tests in this study, a two-tailed (*p* < 0.05) was set as the threshold for statistical significance.

#### 2.3.2 Qualitative data analysis

This study adopted a descriptive phenomenological approach rooted in Husserl’s philosophy, which focuses on the essential structure of lived experience as described by participants. Colaizzi’s seven-step method was selected because it is compatible with descriptive phenomenology and provides systematic procedures for bracketing. First, all audio recordings were transcribed verbatim by two independent researchers within 24 h after each interview. The transcripts were then sent back to the participants to verify the accuracy of the content. Finally, two researchers independently analyzed the transcripts using Colaizzi’s seven-step method:

(1) Familiarization: Researchers repeatedly read the transcripts carefully to achieve a full understanding of the participants’ descriptions.
(2) Identifying significant statements: The transcripts were analyzed line by line to extract meaningful statements related to the research topic.
(3) Formulating meanings: The extracted significant statements were coded and interpreted, during which researchers used bracketing to set aside personal preconceptions. Specifically, each researcher maintained a reflexive journal throughout this step, documenting any emerging assumptions, clinical experiences, or theoretical expectations (including those related to the IMB model). These journals were discussed during biweekly peer debriefing sessions to heighten awareness of potential biases.
(4) Clustering themes: The coded meanings were grouped and merged to form preliminary thematic clusters with similar connotations. Bracketing was maintained to reduce bias from existing theories or assumptions. The two researchers independently clustered themes based solely on participants’ descriptions, without referring to the IMB model at this stage.
(5) Developing an exhaustive description: A detailed and comprehensive description was constructed for each theme, supported by representative verbatim quotations from participants.
(6) Producing the fundamental structure: Similar themes and descriptions were compared, summarized, and refined to extract the core viewpoints and form the final thematic structure.
(7) Seeking verification of the fundamental structure: The final thematic structure was reviewed by the research team to ensure consistency and credibility.

To minimize subjective bias, two additional researchers who were not involved in data collection but experienced in qualitative research independently coded and analyzed the transcripts. Coding results were compared, and any discrepancies were resolved through group discussion until a consensus was reached.

The IMB model served as a sensitizing framework rather than a predetermined coding template. It was introduced only after the thematic structure had emerged from inductive analysis, to facilitate integration with quantitative data and to organize the presentation of findings. Any participant accounts that did not align with IMB dimensions were retained and reported as separate themes.

### 2.4 Ethical considerations

All participants were fully informed about the purpose, procedures, and significance of the study before data collection, and written informed consent was obtained. The questionnaire survey and semi-structured interviews were designed to avoid causing any psychological distress or traumatic recall. All interviews were audio-recorded in full for subsequent verbatim transcription. Audio files were kept confidential by the lead researcher and permanently deleted immediately after transcription. Each participant was assigned an alphanumeric code to protect anonymity and privacy.

This study was approved by the Ethics Committee of Henan Provincial People’s Hospital (Ethics approval number: 2022-041-02). The approval covered the entire study protocol, including both the questionnaire survey and the semi-structured interviews with audio recording. Data collection was conducted between March and July 2025, within the approved validity period.

## 3 Results

### 3.1 Quantitative results

#### 3.1.1 Demographics

A total of 236 stroke patients were investigated in this study, and 224 valid questionnaires were recovered, with a valid recovery rate of 94.91%. Among the 224 patients included in this study, 142 were male and 82 were female. The mean age was 49.49 ± 8.47 years. There were 108 (48.21%) patients with hypertension, 18 (8.04%) with coronary heart disease, and 57 (25.45%) with diabetes mellitus. Detailed information is presented in Table 1.

#### 3.1.2 Univariate analysis

Shapiro–Wilk normality test confirmed normal distribution of nutritional literacy scores across all subgroups. Univariate analyses revealed statistically significant differences in nutritional literacy regarding age, educational level, occupational status, medical payment mode, residential location and monthly per capita household income (all *P* < 0.05). Gender, living arrangement, smoking/alcohol consumption history, disease duration, stroke hospitalization frequency and chronic comorbidities showed no significant between-group differences (*P* > 0.05), detailed statistics are presented in Table 1.

#### 3.1.3 Nutrition literacy score

Total CHI-NLit total scores ranged from 8 to 31 (mean = 18.24 ± 4.85, total scoring proportion = 48.00%). Based on the cutoff of 21.5, 76.3% (*n* = 171) participants had inadequate nutritional literacy, and only 23.7% (*n* = 53) reached qualified level.

Scores of three knowledge-based dimensions were generally higher than skill-based dimensions (average scoring proportion: 49.34% vs. 43.96%). Notably, Food Label and Numeracy was abnormally distributed (median = 0, P25 ~ P75:0–0), with an extremely low scoring proportion of 13.17%; 79.9% (*n* = 179) of all participants gained zero points on this subscale, indicating prominent deficiency in label-related computational ability. Detailed dimensional scores are summarized in Table 2.

#### 3.1.4 Multivariable linear regression analysis

The coding scheme for all independent variables included in the regression analysis is presented in Table 3. All variables with *p* < 0.05 in univariate analysis were entered into a multiple linear regression model. The overall model fitted well (*F* = 12.260, *p* < 0.001), with all VIF < 5 and a Durbin-Watson statistic of 1.847, indicating no significant multicollinearity or residual autocorrelation; the adjusted *R*^2^ was 0.312.

Final regression outcomes demonstrated that middle age (41–59 years, standardized *β* = −0.239, *p* < 0.001) was an independent risk factor for lower nutritional literacy, whereas high school education or above [compared with primary school or below, standardized (*β* = 0.351, *p* < 0.001)] was an independent protective factor. Junior high school education did not reach statistical significance (*p* = 0.067).

After adjusting for age and education, residential location, household income, occupational status, and medical insurance type were no longer statistically significant (all *p* > 0.05), indicating that their associations with nutritional literacy in univariate analysis were largely explained by confounding with education and age. Detailed regression parameters are presented in Table 4.

**Table 4:** Multivariable linear regression analysis of factors associated with nutrition literacy.

Variable	*B*	SE	95% confidence interval	Standardized *β*	*t*	*P*	VIF
Constant	19.016	1.652	[15.760, 22.273]	—	11.509	<0.001	–
Junior high school education	1.375	0.746	[−0.096, 2.846]	0.139	1.843	0.067	1.855
High school and above education	3.51	0.979	[1.581, 5.439]	0.351	3.587	<0.001	3.110
Retired employment status	−1.577	0.860	[−3.272, 0.118]	−0.162	−1.834	0.068	2.527
Employed status	−0.922	0.832	[−2.563, 0.718]	−0.072	−1.108	0.269	1.380
Urban medical insurance	2.79	1.493	[−0.153, 5.732]	0.125	1.869	0.063	1.442
Employee medical insurance	−0.641	0.863	[−2.343, 1.061]	−0.065	−0.743	0.459	2.464
Age (years)	−3.046	0.769	[−4.563, −1.530]	−0.239	−3.96	<0.001	1.179
Current residence	−0.248	0.684	[−1.597, 1.100]	−0.026	−0.363	0.717	1.606
Household per capita monthly income	0.618	0.445	[−0.259, 1.494]	0.101	1.389	0.166	1.705

*R*^2^ = 0.340, Adjusted *R*^2^ = 0.312, *F* = 12.260, *P* < 0.001.

### 3.2 The interview results

Sixteen participants were recruited via purposive maximum variation sampling, covering diverse age, residence, education, income and nutritional literacy levels (11 with inadequate literacy, 5 adequate; 9 males, 7 females). Participant basic information is shown in Table 5. Based on the IMB theoretical framework, three primary themes and eight subthemes were extracted after Colaizzi’s seven-step qualitative analysis.

**Table 5:** General characteristics of participants in the qualitative study.

ID	Age	Gender	Residence	Education	Occupation	Monthly income (¥)	CHI-NLit score	NL level	Stroke duration (months)	Comorbidities
P1	45	Male	Rural	Middle school	Self-employed	≤2000	14	Inadequate	6	Hypertension
P2	52	Male	Urban	Associate	Mid-level manager	4,001–6,000	22	Adequate	3	Diabetes
P3	38	Female	Urban	Bachelor	Civil servant	6,001–8,000	26	Adequate	1	None
P4	58	Male	Rural	Primary	Farmer	≤2000	11	Inadequate	24	Hypertension + Diabetes
P5	35	Male	Urban	High school	Delivery worker	2001–4,000	19	Inadequate	2	None
P6	51	Female	Urban	Associate	Finance staff	4,001–6,000	23	Adequate	4	CHD
P7	48	Male	Rural	Middle school	Farmer	≤2000	12	Inadequate	8	Hypertension
P8	42	Female	Rural	Primary	Homemaker	2001–4,000	15	Inadequate	5	Diabetes
P9	39	Female	Urban	High school	Homemaker	4,001–6,000	18	Inadequate	3	None
P10	44	Male	Urban	Associate	Office clerk	4,001–6,000	24	Adequate	2	Hypertension
P11	33	Male	Urban	Bachelor	Media professional	6,001–8,000	25	Adequate	1	None
P12	56	Female	Rural	Primary	Farmer	≤2000	10	Inadequate	18	Hypertension + Diabetes
P13	41	Male	Urban	Associate	Office clerk	4,001–6,000	21	Inadequate	3	None
P14	49	Male	Urban	High school	Sales manager	4,001–6,000	17	Inadequate	5	Hypertension
P15	47	Male	Urban	Technical secondary	Driver	4,001–6,000	20	Inadequate	4	Diabetes
P16	53	Female	Rural	Middle school	Homemaker	≤2000	13	Inadequate	36	Hypertension

#### 3.2.1 Fragmented information acquisition and barriers to knowledge translation

This theme encapsulates participants’ experiences of accessing and applying dietary information.

##### 3.2.1.1 Information bricolage in the absence of authoritative guidance

Participants uniformly reported that hospital-issued dietary instructions were overly vague and failed to address their individualized needs. This gap forced them to piece together dietary information from diverse non-professional channels, including social media platforms and peer support groups. Nevertheless, the scattered and contradictory nature of these sources triggered severe confusion and uncertainty.

For instance, one farmer commented (P7): “The doctor only advised a low-salt and low-fat diet at discharge, but provided no practical guidance on how to adhere to it. Now I solely rely on short videos—some claim egg yolks should be avoided entirely, while others state three eggs per day is acceptable. I have no idea whom to trust.” Another noted (P12): “Our patient support group shares folk remedies daily. Someone claimed eggplant can absorb fat from blood vessels, and many group members planned to try it.”

##### 3.2.1.2 Culinary skills deficit as a barrier to knowledge translation

Although most participants possessed basic declarative knowledge regarding “what to eat,” they lacked procedural knowledge of “how to prepare” nutritious meals that were palatable, feasible and sustainable. This skills deficit fundamentally hindered the translation of nutritional recommendations into daily dietary practices. For example, a sales manager interviewee stated (P14): “The doctor recommended more whole grains, so my wife purchased brown rice and oats. However, they turned out rock hard after cooking, and no one taught us the proper preparation methods.” Another homemaker interviewee explained (P9): “I know vegetables should be blanched to reduce purines, but blanched vegetables taste tasteless. My husband refused to eat them at all.”

#### 3.2.2 The multifaceted motivational landscape—interplay of responsibility, emotion, and social support

This theme illuminates the complex motivational dynamics governing participants’ engagement with dietary recommendations.

##### 3.2.2.1 Familial responsibility as an endogenous motivational force

For the majority of participants, the primary driver of dietary adherence was not personal health concerns, but a profound sense of familial obligation. A self-employed participant shared (P1): “My son will take the college entrance examination next year. I cannot afford to fall ill. Every time I crave pickled vegetables, I remind myself: if I suffer another stroke, who will take him to the examination?” A finance staff member noted (P6): “My mother is over 80 years old. I cannot let her become my caregiver. I must stay healthy enough to walk independently and avoid burdening her.”

##### 3.2.2.2 Negative affect as a trigger for behavioral lapses

Negative emotional states—including frustration, anger, anxiety and exhaustion—frequently led to temporary abandonment of dietary regimens. Participants were clearly aware of this pattern yet struggled to exert self-regulation during emotionally charged moments. A delivery worker stated (P5): “I received a customer complaint and was fined 200 yuan that day. I returned home exhausted and had no energy to cook. I opened the fridge, found leftover braised pork from the previous day, and ate it standing up without reheating. I blamed myself while eating, but I could not stop.” Another homemaker interviewee said (P8): “My husband made sarcastic remarks, saying I was being picky after falling ill. I got so angry that I went out, bought fried chicken and ate it all. I cried afterwards—I felt foolish for punishing my body to retaliate against him.”

##### 3.2.2.3 The dual effects of family support

Family support acted as a double-edged sword, either strengthening or undermining dietary adherence based on its form and delivery. Active, collaborative support boosted motivation, whereas well-meaning but inappropriate care inadvertently sabotaged dietary efforts. A farmer participant commented (P4): “When my children come to visit, they always say ‘Dad cannot eat anything now, poor thing’ and insist on adding meat to my bowl. If I refuse, I ruin the family atmosphere; if I accept, I feel guilty afterwards.” A driver shared (P15): “My wife follows the same low-fat diet with me. She jokes, ‘If we are going to face health issues, we’ll face them together.’ Having a companion makes it much easier to persist.”

#### 3.2.3 Behavioral skills deficits—the chasm between cognition and execution

This theme identifies critical gaps in the practical competencies required for effective dietary self-management.

##### 3.2.3.1 Pervasive inability to interpret food labels

Participants exhibited profound deficits in understanding and applying food label information, particularly the calculation and utilization of Nutrient Reference Values (NRV%). This competency gap represented the most prominent weakness in nutritional skills. An office clerk stated (P10): “After learning to read ingredient lists, I found that the so-called ‘0-fat’ yogurt I used to enjoy contains more sugar than cola. Now I feel I’ve finally learned to identify deceptive food labels.” A civil servant noted (P3): “Initially, I could not understand NRV% at all. A nurse in the nutrition clinic taught me step by step, and then I mastered the calculation method.”

##### 3.2.3.2 The intractable constraint of workplace socializing

For employed participants, occupational social obligations—especially business dinners involving alcohol and high-fat foods—posed an unavoidable challenge to dietary adherence. Socio-cultural pressures routinely trumped health rationality, creating a painful conflict between medical requirements and professional expectations. A mid-level manager explained (P2): “When a client proposes a toast, can I refuse by saying ‘I’m ill and cannot drink’? That would ruin the business atmosphere. So I drink, then go home and secretly take an extra pill. This inner conflict is unbearable.” A media professional shared (P11): “Working until 9 p.m., the only takeout options available are fried chicken and barbecue. I know it’s unhealthy, but when I’m starving with stomach pain, I cannot care about dietary rules. I fill my stomach first and regret later.”

##### 3.2.3.3 The dynamic evolution of self-efficacy

Participants’ self-efficacy—confidence in sustaining dietary changes—evolved in a spiral process shaped by successive successes and failures. Positive feedback loops enhanced perceived control, while repeated failures risked inducing learned helplessness. An office clerk stated (P13): “After 3 months of consistent healthy eating, all my lipid indicators returned to normal! Holding that laboratory report, I felt capable of anything. Now I’m truly interested in nutrition knowledge—I feel in control of my health.” A homemaker shared (P16): “The most devastating moment was after 6 months of strict dietary restrictions: I lost no weight, and my lipid levels were even higher. I stood in the kitchen for a long time, then took out a bottle of peanut oil and fried a plate of spring rolls, eating all of them. I thought: it makes no difference anyway, so I might as well enjoy myself.”

### 3.3 Integration of quantitative and qualitative findings

Findings from the quantitative survey and qualitative interviews were integrated using a joint display framework (Table 6) following a sequential explanatory mixed-method design. Based on the three domains of the IMB model, the joint display yielded three types of meta-inferences: complementary, consistent, and expansive.

**Table 6:** Integrated summary of quantitative and qualitative findings based on the IMB model in mixed-method research.

IMB dimension	Quantitative findings	Qualitative interview evidence	Integrated meta-inferences (Complementary/Consistent/Expansive)
Information domain	76.3% of participants scored below the cutoff of 21.5 for nutritional literacy, with only 23.7% achieving the qualified level 79.9% obtained zero points in the food label calculation test	• Vague inpatient education: “Doctors only told me to eat low-salt and low-fat food” (P7) • Limited understanding of labeling: “I could not understand NRV% at all at first” (P3)	Complementary: Quantitative data quantified the overall prevalence of insufficient nutrition knowledge; verbatim qualitative quotations further explained underlying contributors, including oversimplified hospital nutrition education and misleading commercial food labeling.
Motivation domain	Middle-aged (41–59 years), rural residents and manual laborers exhibited significantly lower nutritional literacy scores (*P* < 0.05)	• Workplace constraints: “I cannot refuse to drink during business toasts with clients” (P2) • Fragmented information access: “We learn nutrition knowledge from online influencers on short-video platforms” (P12)	Consistent: Quantitative analyses identified high-risk vulnerable subgroups; independent qualitative findings confirmed that geographic and occupational barriers restrict individuals’ willingness to adopt healthy eating, verifying the quantitative results mutually.
Behavioral skill domain	Only 50.5% of participants passed the household food portion measurement assessment	• Practical cooking barrier: “Brown rice always turns out too hard to eat” (P14) • Taste-related non-adherence: “Blanched vegetables taste bland and unpalatable” (P9)	Expansive: Quantitative results objectively demonstrated poor practical dietary performance; qualitative evidence further expanded targeted intervention recommendations, such as implementing hands-on cooking training sessions to improve long-term dietary adherence.

P = participant number of original interview transcripts.

#### 3.3.1 Complementary integration (information domain)

Quantitative findings revealed that 76.3% of participants had inadequate nutritional literacy, with 79.9% scoring zero on the food label calculation test. Qualitative data supplemented concrete causes for this deficiency. Participants consistently reported that hospital discharge instructions were overly simplified (“Doctors only told me to eat low-salt and low-fat food,” P7) and that they resorted to fragmented online sources (“We learn nutrition knowledge from online influencers on short-video platforms,” P12). Together, these quantitative and qualitative findings complement each other: the former quantifies the prevalence of information deficiency, while the latter explains its underlying contributors.

#### 3.3.2 Consistent integration (motivation domain)

Quantitative univariate analyses identified middle-aged patients, rural residents, and manual laborers as vulnerable subgroups with significantly lower nutritional literacy scores. Qualitative data corroborated these disparities from two angles: occupational social pressure (“I cannot refuse to drink during business toasts with clients,” P2) and the limited information environment in rural areas. Importantly, multivariate regression further revealed that these disparities were mediated by education and age rather than serving as independent predictors. Thus, the qualitative evidence provides convergent validation of the quantitative subgroup differences, while the regression analysis clarifies the underlying mediating mechanisms.

#### 3.3.3 Expansive integration (behavioral skill domain)

Quantitative data showed that only 50.5% of participants passed the household food portion measurement assessment, indicating suboptimal practical dietary skills. Qualitative findings expanded beyond this numerical finding by revealing specific, actionable obstacles. Participants reported practical cooking difficulties, such as improper preparation of coarse grains (“brown rice and oats turned out rock hard after cooking,” P14) and palatability issues (“blanched vegetables taste tasteless,” P9). Conversely, one participant noted that targeted education was effective (“A nurse taught me step by step how to calculate and choose yogurt,” P3). These qualitative insights expand the quantitative results by identifying not only that skills are deficient, but also which specific skills need to be targeted and how interventions might be designed (e.g., hands-on cooking training, food label education).

## 4 Discussion

### 4.1 Overall insufficient nutritional literacy necessitates targeted improvement

In this mixed-method study, the mean nutritional literacy score of young and middle-aged patients with ischemic stroke was 18.24 ± 4.85, representing an overall scoring proportion of only 48.0%. Moreover, 76.3% of participants failed to reach the qualified cutoff of 21.5 points. In contrast, a study by Zhang et al. (28) reported that nutrition literacy in patients with type 2 diabetes mellitus was at an upper-middle range. The substantially lower level observed in our stroke population (scoring proportion: 48.0%) may be attributed to the sudden onset of stroke and the lack of systematic post-discharge nutrition education in this population.

Several contextual characteristics of enrolled subjects contributed to this unsatisfactory outcome: nearly half of participants resided in rural areas, over 60% had education below senior high school level, and most had limited household disposable income; meanwhile, most patients were newly diagnosed with ischemic stroke and lacked systematic long-term nutrition education after hospitalization. Existing literature has confirmed that improved nutritional literacy effectively optimizes daily dietary structure, controls blood lipid, blood glucose and other vascular risk indicators, and further facilitates secondary prevention against stroke recurrence (29). Given our findings, routine nutritional literacy screening can be incorporated into admission assessment for young and middle-aged stroke inpatients, and individualized health education should be formulated in accordance with patients’ socioeconomic features.

### 4.2 Severe deficit in practical dietary skills constitutes the primary bottleneck

This study identifies a critical bottleneck in the pathway from healthy eating attitudes to dietary behaviors: although positive attitudes are necessary prerequisites, severe deficits in practical dietary skills—particularly nutrition label use—constitute the primary barrier to behavioral translation. Our findings complement those of Graham et al. (30), who demonstrated that nutrition label use partially mediates the attitude–behavior relationship. While their study showed that label use serves as a behavioral pathway, our results further reveal that this mediating pathway fails to initiate precisely due to a widespread lack of label-use skills.

Graham et al. (30) found that among college students, frequent nutrition label use was associated with healthier dietary intake. However, their study implicitly assumed that participants possessed basic label-reading and numeracy skills. In contrast, our quantitative data showed that 79.9% of participants scored zero on the Food Label and Numeracy subscale, indicating that the vast majority could not effectively interpret nutrition facts panels or calculate percentage of nutrient reference values. Qualitative evidence further clarified that although patients knew they should choose whole grains and limit added sugars, they could not locate key information on food labels or translate it into purchasing decisions. For example, one participant stated, “I know whole grains are healthy, but I do not know how to cook them to taste good, so I stopped eating them”—a vivid illustration of the knowledge–behavior gap. From the perspective of the Knowledge-Attitude-Practice (KAP) theory (31), health knowledge alone cannot automatically translate into sustained healthy dietary behaviors unless supplemented with actionable skill training.

Therefore, future nutrition interventions should shift from knowledge-only approaches toward skills-based empowerment. Specific recommendations include: (1) scenario-based label reading training using real food packages; (2) simple home cooking demonstrations and hands-on practice for healthy but less familiar foods (e.g., coarse grains, legumes); and (3) inclusion of skill assessments in intervention evaluations. In conclusion, healthy eating attitudes provide the “engine,” while practical skills such as nutrition label use provide the “steering wheel and accelerator”—both are required to truly bridge the knowing–doing gap.

### 4.3 Low education and middle age, rather than rural residence or low income *per se*, are the fundamental determinants of inadequate nutritional literacy

Univariate analysis showed that middle age, low education, rural residence, farming/manual occupation, and low household income were significantly correlated with lower nutritional literacy. However, after adjustment for confounding indicators via multiple linear regression, only education level (*β* = 0.351, *p* < 0.001) and age (*β* = −0.239, *p* < 0.001) retained independent statistical significance, whereas residential location, household income, occupational status, and medical insurance type became non-significant (all *p* > 0.05). This indicates that education and age act as core confounding or mediating factors: rural residents tend to have lower educational attainment and household income, and these characteristics are highly clustered among middle-aged adults with limited education. After controlling for education and age, the explanatory power of other socioeconomic variables was absorbed, suggesting that education level is the most fundamental social determinant of nutritional literacy in this population, while age reflects the declining cognitive reserve and information acquisition capacity associated with aging.

From a methodological perspective, this finding carries two important implications. First, it prevents the misinterpretation of superficially correlated factors as independent causal factors. Had the analysis relied solely on univariate results, researchers might have mistakenly targeted “rural residence” or “low income” as direct intervention targets while overlooking the more fundamental issue of educational deficits. Second, it directs intervention strategies toward modifiable core factors. Although place of residence and income are difficult to change in the short term, educational disadvantages can be compensated for through health literacy-tailored interventions, such as visually simplified educational materials and low-literacy-burden dietary guidance. Encouragingly, recent intervention evidence supports the feasibility of such approaches: a structured nutrition education program among low-income Saudi families—a population with comparable educational disadvantages—demonstrated significant improvements in both food literacy (mean score increased from 46.4 ± 10.9 to 58.4 ± 7.1, Cohen’s *d* = 1.32) and dietary intake following the intervention. This suggests that even among populations with low baseline education levels, targeted, skills-based nutrition education can effectively enhance nutritional literacy (32). Similarly, age-related capacity limitations can be addressed through time-friendly, low-execution-burden intervention formats, such as micro-learning modules or family-based meal preparation training.

### 4.4 An IMB model-based intervention framework derived from integrated evidence

Based on the complementary, consistent, and expansive integrated findings across the Information, Motivation, and Behavioral Skills domains (Table 6), we propose a multi-level intervention strategy grounded in the IMB framework. This framework is conceptually aligned with the comprehensive health literacy model proposed by Sørensen et al. (33), which defines health literacy as involving knowledge, motivation, and ability to access, understand, appraise, and apply health information. Rather than repeating specific intervention tactics already discussed in previous sections, this section synthesizes the integrated evidence into a coherent, theory-driven intervention framework.

#### 4.4.1 Information dimension: standardizing authoritative information supply

Patients’ nutrition information acquisition was characterized by vague in-hospital dietary instructions and conflicting online nutrition rumors (complementary integration). To address this, hospitals should establish a linked hospital-community-family continuous nutrition education system. Clinical nurses should deliver visualized, plain-language dietary advice during hospitalization using intuitive measurement descriptions (e.g., “one fist of staple food, one palm of protein”). After discharge, authoritative dietary content should be regularly disseminated through official medical platforms to help patients identify false online nutrition information.

#### 4.4.2 Motivation dimension: activating internal motivation and optimizing family support

The consistent integration revealed that family responsibility was the primary endogenous motivation facilitating dietary compliance, whereas negative emotions triggered by work setbacks and family conflicts were major inducements of unhealthy dietary relapse. Furthermore, family support produced dual-sided effects on dietary management. Hence, interventions should: (1) reinforce patients’ sense of responsibility for family health; (2) provide emotional management training (e.g., the “3-min delay rule”—when craving unhealthy food, drink a glass of water and wait 3 min before deciding); and (3) involve family caregivers in health education to prevent excessive indulgent feeding and promote shared healthy eating practices. Furthermore, evidence from other populations suggests that tailoring interventions to individuals’ preferences and social contexts can enhance motivation and adherence (34). Such insights may inform the design of personalized motivation strategies for stroke patients, although further validation in this specific population is needed.

#### 4.4.3 Behavioral skills dimension: bridging skill gaps and empowering self-management

The expansive integration identified three prominent skill deficits: incomprehension of food labels, workplace banquet-related social dilemmas, and fluctuating self-efficacy. Corresponding interventions should target these deficits through: (1) case-based training on nutrition label reading and NRV% calculation using real food product packages; (2) simulated social dining scenarios to teach polite refusal and healthy substitution skills; and (3) a periodic short-term health goal feedback mechanism (e.g., weekly 10-min phone check-ins during the first month post-discharge, self-monitoring logs) to create continuous positive feedback and prevent learned helplessness. Crucially, the implementation of these skills-based strategies should be embedded within a multidisciplinary care framework. Liu et al. (35) identified nutrition education as a key modifiable factor influencing nutrition literacy in stroke patients, emphasizing the need for targeted, ongoing educational interventions. Similarly, Mullins (36) demonstrated that a multidisciplinary approach—encompassing nutrition screening, individualized medical nutrition therapy, and continuous monitoring—is essential for improving post-stroke nutrition management outcomes.

### 4.5 Limitations of this study

This study has several limitations. First, participants were recruited from a single tertiary hospital in Zhengzhou using convenience sampling, which may limit the representativeness and generalizability of the findings to broader stroke populations. Nutritional literacy levels may vary across different geographic regions and healthcare settings, and our single-center design cannot capture such diversity. Second, the cross-sectional design precludes establishing causal relationships between nutritional literacy and its associated factors, and cannot capture longitudinal changes in nutritional literacy over time.

Third, the qualitative findings rely on patients’ subjective self-reports, which may introduce reporting bias, such as recall bias or social desirability bias. Additionally, although data saturation was achieved, the interpretation of results may be influenced by the researchers’ experience and judgment. Fourth, the CHI-NLit cutoff value of 21.5 points has not been validated in ischemic stroke patients and was used exploratorily in this study, which may lead to potential classification bias. Future studies should establish stroke-specific cutoff values for this scale.

Fifth, several clinically relevant variables—including functional status (mRS), nutritional risk (NRS-2002), previous dietitian consultation, and discharge nutrition education—were not collected. These indicators may influence patients’ nutritional literacy and dietary self-management. Future multicenter prospective studies incorporating comprehensive clinical indicators are warranted to systematically identify core determinants of nutritional literacy in stroke patients. Additionally, longitudinal and randomized controlled trials are needed to evaluate the long-term effects of IMB model-based interventions on nutritional literacy and clinical outcomes.

### 4.6 Conclusion

This sequential explanatory mixed-method study revealed that nutritional literacy among young and middle-aged patients with ischemic stroke is generally insufficient, with particularly severe deficits in practical skills (e.g., food label interpretation, household food measurement). The quantitative findings identified low education and middle age as the fundamental determinants, while qualitative findings further explained the underlying mechanisms: fragmented information access, workplace social constraints, negative emotional triggers, and fluctuating self-efficacy. The integrated analysis, guided by the IMB model, generated targeted intervention strategies across information, motivation, and behavioral skills domains. These findings may inform the design of future interventions to improve nutritional literacy in this population. However, further longitudinal and interventional studies are needed to determine whether improving nutritional literacy reduces stroke recurrence or improves clinical outcomes.

## Data Availability

The datasets presented in this article are not readily available because the qualitative data, including interview transcripts and audio recordings, are not publicly available due to the presence of potentially identifiable and sensitive personal information, and because participants did not provide consent for public data sharing. Requests for access to the quantitative data should be directed to GZ. Requests to access the datasets should be directed to GZ, zgf1979063863@163.com.
